# Vitality and Immunity

**Published:** 1903-03

**Authors:** Carl Schulin

**Affiliations:** Billings, Mont.


### VITALITY AND IMMUNITY.

By Carl Schulin, M.D.,

BILLINGS, MONT.

(Continued from page 88.)

The neurin bases are derivatives of protagon and the lecithins, which may be found everywhere in the tissues. They are neurin, cholin, betain, muscarin, etc. Neurin has been found in the brains right after death. It is a strong heart poison. Cholin has been found in most glands. It is also a poison, but not so strong as neurin. All the neurin bases can, by oxidation, be changed to less toxic compounds.

Bouchard was the first to investigate the toxicity of the urine. He maintains that a man in about fifty hours excretes through the kidneys a quantity of poison sufficiently large to kill himself with. There are all kinds of poisons in the urine—narcotics, sialagogues, myotics, and such as produce paralysis or convulsions. The urine which is secreted in daytime is more narcotic; the night urine is more conducive to spasm. Bouchard also found that the toxicity of the urine can be decreased by inhalation of oxygen.

Gautier discovered first that only four-fifths of the oxygen excreted by the body originates from inhaled oxygen. He already expressed the opinion that there might be a ferment in the body which would furnish the last fifth from some other source. In 1892 Poehl discovered in the spermin a ferment which doubtless does what Gautier anticipated.

Substances of especially great toxicity are found in the urine during attacks of contagious diseases. Griffith found here some well-characterized alkaloids in sufficiently large quantities to enable him to study not only their chemical composition, but also their physiological action. Some of them are also found in pure cultures of the germs which cause the disease. They are called ptomaines. Poehl puts them together with the leucomaines; there is, however, a characteristic difference between both. The ptomaines can also, by oxidation, be changed to less poisonous substances.

Very strong leucomaines are found in the urine in cases of cancer, eczema, epilepsy, etc. Thomas Oliver, in the preface to his translation of Bouchard's *Lectures on Auto-intoxication in Disease*, says that the urine of maniacs, when injected into the blood of animals, produces excitement and convulsions, while that of melancholics causes depression of spirits.

Bouchard himself says in the above-named work: "One day of great muscular activity, spent in the open air in the country diminishes the toxicity of the urine by one-third, and on that day the toxicity does not diminish only during the time devoted to muscular exercise. The toxicity which diminishes during work, remains less during the repose which follows this work and during the sleep which succeeds this day of muscular activity."

Bouchard, in the writer's opinion, emphasizes too much the muscular activity and overlooks entirely the part of the light. Muscular exercise in open air and sunlight brings the blood into circulation and assists it in taking up oxygen and excreting the waste products. The same exercise, however, in spite of the best air, will have a different effect in a dark hall. It is not the muscular activity and the oxygen alone that we need. We also need the sunlight with its actinic rays for the production of spermin. The sun does more for the reduction of the toxicity of the urine than the labor.

Labor and fever have the feature in common that both cause an increase in the consumption of albuminous substances through metabolism. The result, however, is entirely different. Labor is a normal function of the body. If performed in pure air and good light, it leads to the production of large quantities of urea and few leucomaines. It stimulates the production of spermin. Therefore, the urine will be only slightly toxic and will remain so, because the increase in spermin will outlast the labor.

Fever is not a normal function of the body. It is a reaction against a disease. The disease produces a great waste of albuminous matter in the body, and this waste leads, not so much to the production of compounds of the kreatin group, as of such of the xanthin group and the neurin bases. The result will be an excessive formation of uric acid instead of urea. All conditions are unfavorable to the acquisition and the work of oxygen, and the urine will be charged with leucomaines. The amount of urea will be also increased, but the other compounds so much the more. Conditions are also unfavorable to the production and the work of spermin, and death is liable to follow from auto-intoxication.

Not long ago, Gautier, by careful investigations, corroborated what Schoenbein, Hilger, and others assumed years previously, namely, that besides processes of oxidation also processes of reduction take place simultaneously in the tissues. The latter occur in and around the nucleus, the former in the protoplasma.

Normally, the cells are immersed in oxygen-bearing juices and live aërobically. Gautier, however, showed that they can also live facultative-anaërobically and the products of the anaërobic life are only quantitatively different from those of the aërobic life. The former contain about three times more leucomaines than the latter, and at the same time the juices show acidulation. The distinction between aërobic and anaërobic life is liable to create the wrong impression that only the former was based on assimilation of oxygen. The difference, however, is only in the source of the oxygen. In aërobic life oxygen which comes directly from the air is consumed, while in anaërobic life the oxygen is,through processes of reduction, extracted from the tissues. Therefore the presence of free oxygen protects albumin against decomposition by anaërobic saprophytic germs, as it is doubtless easier to absorb free oxygen than to extract it. At the same time free oxygen kills the germs, because the products of decomposition which are their food are not formed.

The action of spermin is not limited to nitrogenous substances. It operates as well on fat, fatty acids, carbohydrates, aromatic compounds, etc. The acidulation of the juices is caused especially by lactic acid. The active spermin also destroys this and restores the alkalinity of the juices.

It has been satisfactorily proven by Kossel and others that the xanthin compounds and uric acid originate from nucleo-albumin and not from the albumins of protoplasma. In retrogressive metamorphosis the nucleo-albumin is first split into albumin and nuclein; the latter is split into albumin and nucleinic acid, and this into phosphoric acid, xanthin compounds, carbohydrates, and spermin. The reader will notice that the nucleo-albumin has a very much more complicated structure than the albumins of protoplasma, several molecules of which it contains.

If the juices are alkaline and contain a sufficient amount of oxygen, the xanthin compounds, under co-operation of the spermin, are oxidized first to uric acid and then to urea, and the carbohydrates are oxidized to carbonic acid and water. Urea is the highest stage to which nitrogenous matter is oxidized by the metabolism.

If the juices, however, are neutral or slightly acid and short of oxygen, spermin will crystallize as insoluble and inert phosphate, and the xanthin compounds as well as the carbohydrates, etc., will remain more or less unchanged. A condition of retarded metabolism will result which Hippocrates already, with admirable acumen, distinguished as "diathesis" from "dyscrasia." Following him, still, to-day, we speak of uric-acid diathesis and of scrofulous dyscrasia.

A large number of diseases belong under the head of diathesis which have the peculiarity in common that they impress us with being symptoms of deeper-seated diseases rather than diseases of their own. Two main groups are formed by the question whether the metamorphosis of the nitrogenous or that of the non-nitrogenous substances is the more retarded. To the latter group belongs, for instance, glycosuria. While many cases are well pronounced, there is often no distinct line to be drawn between the members of both groups, nor between them and the auto-intoxications. We speak of auto-intoxication when the toxic products of retarded metabolism prevail, and of diathesis when the non-toxic prevail. The relation between diathesis and dyscrasia is causal. Dyscrasia causes diathesis, and a diathesis from another source is a predisposing moment to infection, and consequently development of a dyscrasia.

As a gauge for the intra-organic oxidation, Poehl introduced the following coefficient: First, the quantity of all nitrogen in a certain measure of urine is found, and then the nitrogen which is contained in urea. Poehl gave to the proportion between both the name of "coefficient of oxidation." Normally, 91 to 94 per cent. of the nitrogen eliminated by the kidneys is contained in the urea. Sometimes Poehl found even 96 per cent. of all nitrogen in urea.

A lowering of the coefficient under 91 per cent. indicates a decrease of the intra-organic oxidation below the normal. Poehl calls this decrease moderate until it reaches 87 per cent., and below this he calls it serious. The coefficient may sink below 60 per cent. In a case of Asiatic cholera Poehl found once a decrease to 47.8 per cent. In all feverish diseases it is seriously decreased, and if death comes it is actually caused by autointoxication.

Poehl's coefficient is of the greatest value in detecting the earliest beginning of chronic diseases, especially in childhood. Early recognition is one of the chief tasks of preventive medicine.

Certain kinds of cases occur everywhere looking exactly alike. So every reader will once in a while meet with a well-dressed little girl "of good family," about ten years old. She looks as healthy and bright as a child of her age can be. The only thing that you observe is that she wears spectacles. She states that as soon as she takes off her glasses she suffers a headache. The mother states that the little girl has never been sick. "She is only a little delicate, as I am myself." The mother thinks children "of good family" are of course not so robust as farmer boys. If you inquire further you will find that "delicate" means repeated but slight attacks of nasal and bronchial catarrh, headache, slight fever, and costiveness. Besides these symptoms you will find a very light degree of anæmia and here and there an enlarged lymphatic gland. If you examine the urine of such a little girl after Poehl's method you will always find the coefficient of oxidation moderately lowered.

If you place such a little girl under the same conditions as the farmer boy—that is, if you make her work in fresh air, climb hills, ride horseback in bright sunshine, etc.—then after a few months she will come home with red cheeks and without spectacles. The headaches have disappeared, and when she lost her spectacles while riding horseback she discovered that she could do as well without them. Her complexion may have suffered a little, but how much did she gain for all her future life.

If, on the contrary, such a little girl is kept indoors to protect her from catching cold; if, whenever she leaves the house, her face is protected against the sun with a veil to preserve the complexion, the outcome will be different.

The ideal of preventive medicine is not only to protect and prolong the life of man, but even to make it everlasting; to give us earthly, not heavenly, immortality. For practical reasons, there is little hope for our ever reaching this end. Nevertheless, through Pasteur's and Brown-Séquard's genius, we have gained two important points: First, we have recognized the real cause of death from senility; second, we have found many new means at least of prolonging life, if we cannot perpetuate it.

We know to-day that flesh decays after death, not from intrinsic causes, but by the invasion of germs; and to-day we know that life is terminated in a similar way. Senility is not a natural consequence of time; it is a disease. It is the most chronic form of septicæmia. Germs conquer our bodies by degrees, consuming their vitality. On account of the practical impossibility of keeping them away forever, we all finally have to succumb to them. We succumb either to a last virulent attack or fade away and die the peaceful death of heart-failure from auto-intoxication.

Therefore, preventive measures should be taken from the very first beginning of life. They should begin during fœtal life, even before fecundation. People who are to become parents should remember that only healthy people can beget healthy children.

The most insidious of all germs is the tubercle bacillus. We must be on the lookout for it from the earliest youth. In childhood the epidermis is thin and vulnerable, and slight abrasions lead sometimes to the formation of warts. In such warts, besides other germs, tubercle bacilli have been found. Beside the skin and the intestinal canal, the respiratory tract will furnish places of entrance.

The bacillus of human tuberculosis, like the gonococcus, has not yet been found outside of the human body, except where it apparently came from people affected with the disease.

Human tuberculosis is more or less transmissible to other mammalia, but not to birds. Birds, however, have their own tuberculosis. Their tubercle bacillus is different from that of man. Cattle and swine have a tuberculosis of their own as well. Here the question arises whether the tubercle bacilli of different animals are different, well-defined species or only one specie, certain features of which have changed by adaptation to new conditions. This question is now prominent since the address which Koch delivered at London before the Tuberculosis Congress in July, 1901, in which he declared it unnecessary to take any preventive measures against bovine tuberculosis. Before the discovery of the tubercle bacillus, on Virchow's authority, human and bovine tuberculosis were considered as two entirely different diseases. The latter was called grape disease (Perlsucht). In 1884, Koch pronounced both diseases to be identical. He said they both were caused by the same bacillus and the only difference was in the host. His authority was so great that he even outranked Virchow, and his opinion was generally accepted as correct. Virchow never admitted defeat. He always protested against Koch's claim, and soon others contended that the tubercle bacilli of different animals showed some marked differences. This was especially done in France and Italy. Koch soon admitted the existence of slight differences, but was led to a radical change of opinion only when he found that it was impossible to inoculate human tuberculosis into cattle. He could, of course, not make the counter-experiment from cattle to man. He only inferred that this would also be impossible from the fact that children which had been fed with milk containing bovine tubercle bacilli hardly ever develop tubercular lesions in the mucous membranes of the bowels. Consequently, he pronounced bovine tuberculosis as non-transmissible to man and preventive measures against it as unnecessary.

The writer cannot agree with Koch in two points:

First, it seems to him that the impossibility of an acute transmission of the bacilli *en masse* does not exclude the possibility of a slow, gradual transmission, during which some of the germs gain time to adapt themselves to the new host. If a child drinks for months every day milk containing the bovine tubercle bacillus, the character of the infection will be quite different from that by the hypodermic injection of a pure culture.

Second, the writer cannot admit tubercular ulcers as primary lesions from the infection with the bacilli. If the bacilli are introduced with milk probably no primary lesions at all will be made in the bowels when some of them are absorbed. The first lesions must be looked for not in the bowels, but in the mesentery glands, and really, what our medical forefathers called "tabes mesenterica" is a local tuberculosis of the mesentery glands without lesions of the mucous membrane!

Max Wolff (*Deutsche medicinische Wochenschrift*, 1902, No. 32) also found that tubercle bacilli, when introduced with food, pass through the intact mucous membrane of the bowels and make primary lesions at distant places, especially the mesentery glands. He also states that Ostertag in thousands of hogs affected with tuberculosis, which they contracted with food, never found any lesions in the bowels, but always in the lymphatic glands.

In the same article Wolff reports the result of a post-mortem on a man of sixty-three years, who suffered more than a year with diarrhœa and pains in the abdomen. There were several apparently old ulcers in the mucous membrane of the bowels, together with fresh miliary tuberculosis of the peritoneum and spleen, while the lungs and bronchial glands were intact. Wolff says nothing about the condition of the mesentery glands, still, he takes this case for one of primary tuberculosis of the bowels, according to Koch. He succeeded in transferring tuberculosis from this case on guinea-pigs and from them on a calf which developed all the characteristic symptoms of regular bovine tuberculosis (Perlsucht).

The tubercle bacilli, having passed through the epithelial cover of the body, first settle in lymphatic glands where they may remain for years, apparently without doing any harm. Here is the place where bovine tubercle bacilli might be transformed into human tubercle bacilli. The writer would not be surprised if, in the course of time, we would discover that other (saprophytic) bacilli can do the same.

The most conspicuous symptom which the tubercle bacilli produce in the lymphatic glands is a non-inflammatory enlargement. In children this is named scrofulosis. Such children are generally not considered as sick; they go to school. As a rule, they have some slight troubles—a little eczema, some nasal and post-nasal catarrh, catarrh, or even suppuration of the middle ear, sore eyes, etc. Beside they may have two different kinds of habitus. They may be either too fleshy and slow in their actions, or they may be too lean, with a pale, thin skin through which the blue veins are plainly visible, and they are at the same time bright and exceptionally intelligent. If the urine of such children is examined after Poehl's method, the coefficient is always found more or less below the normal. The physicians of former ages would say the "vitality" of such children is lowered; they have a dyscrasia. When such children are wounded the wound does not heal so well as in healthy children.

When people affected with local tuberculosis, in the lymphatic glands or elsewhere develop general tuberculosis, the question arises whether this comes from the old germs or from a new infection. It is impossible to decide this question directly by facts. The reader will have no doubt that a new infection could accomplish the result, but he will find it difficult to explain why the first did not accomplish it altogether. The writer asks: Why does tuberculosis so often remain local for years, with a pronounced tendency toward self cure, while in other cases it suddenly becomes virulent and spreads all over the body?

The tubercle bacilli, by taking possession of the lymphatic glands and by lowering the vitality, make the body defenceless against other infections. The tubercle bacilli do not, like the typhoid bacilli, immunize their host against further infection with the same germ. They even seem to predispose the body to infection with all kinds of bacteria.

Baumgarten, who divides with Koch the honors of having discovered the tubercle bacillus, found that it has no positive chemotactic force. It has a peculiar influence on the fixed connective-tissue cells, promoting karyokinesis. It causes these cells to increase in number and gather around it. Thus the tubercle originates. The leucocytes do not put in an appearance until later on, apparently attracted by something else than the tubercle bacilli. It seems that the latter take up oxygen from the tissues and kill locally by textural asphyxia. A striking feature is the small number of bacilli compared with the quantity of tissue they kill. They do their work with their metabolism, not with chemotaxis. Therefore, the actinic rays of light are their worst enemies. They stimulate chemotaxis; they attract the leucocytes; they cause spermin to be produced—and the tubercle bacilli succumb. This accounts for Finsen's success with phototherapy in local tuberculosis, and here we see a great future for the treatment of tuberculosis generally.

Koch, in 1890, found that in guinea-pigs, after inoculation with pure cultures of tubercle bacilli, the wound usually heals rapidly, and no trace of the operation is left except an enlargement of the nearest lymphatic glands. About two weeks later, at the point of inoculation, an induration appears which ulcerates and remains so until the death of the animal. If, however, another inoculation is made before the first wound heals, this will prevent it from healing. The surrounding tissues assume a dark color and necrosis sets in. The tubercle bacilli act like anthrax bacilli, apparently with negative chemotactic force. After the necrotic parts have sloughed away a sound ulcer remains, which heals rapidly without leaving any enlargement of the lymphatic glands behind. The same result was obtained with dead cultures of the tubercle bacilli or with the toxins alone. The toxins are harmless to healthy animals, but very poisonous to such as contain tubercle bacilli.

Koch found that if the material for the further injections was more diluted and the injections were repeated about once a day, the animals improved. Instead of dying in from six to ten weeks, they lived as long as nineteen weeks. Thereupon Koch based his hope that it might be possible to prolong the life of tuberculous subjects by the hypodermic injection of tubercle toxin. For this purpose he prepared his famous tuberculin, which is a 50 per cent. glycerin extract of pure cultures of tubercle bacilli. It contains only the toxin, not the bacilli.

The reader knows what a disappointment followed its use. Instead of prolonging it seemed to shorten the life of tuberculous patients. Its use on men was soon abandoned, apparently by everybody. It remained in use only in veterinary practice, for the detection of tuberculosis in cattle.

Koch, however, remained unmoved. He insisted on his being on the right road to the detection of the cure for tuberculosis, and tried to improve his tuberculin. Finally, he found a preparation which he called T. R. The difference between this and the old tuberculin is that the latter is a protein, while T. R. is a plasmin, simply the expressed juice of the bacilli. Koch claims to have immunized and cured guinea-pigs with it. His claims, however, were hotly contested, especially by Baumgarten.

However much Koch's treatment was discredited, it has some advocates among men of established authority, for instance, Klebs and others.

Fisch (*Journal of the American Medical Association*, October 30, 1897) succeeded in immunizing horses against tuberculosis with Koch's old tuberculin, as well as with T. R., and used afterward their blood serum for curative purposes. So far he did this only with guinea-pigs, but he claims to have obtained good results.

In 1901, Koch found an advocate who bids fair to turn the tide again in his favor. Goetsch was one of the first who experimented with tuberculin, in 1891. With rare acumen he avoided the mistake by which others brought the tuberculin into discredit. While others injected more tuberculin the more fever the patient had, he recognized early the danger threatening from this side, and used it only in doses so small as to avoid a rise of temperature. The diagnosis of tuberculosis was based on the rise of temperature after larger doses of tuberculin and on the presence of the bacillus in the sputum.

Of 175 patients whom he succeeded in keeping more than four weeks under treatment, Goetsch cured 125, or 71 per cent. The remaining 50 patients mostly gave up the treatment too early, after having been improved but not fully cured. The average duration of the treatment in the cured cases was 198 days, varying from 50 to 791 days. The average increase in weight was 19 pounds.

Goetsch commences the treatment with doses of 0.0001 and increases slowly. He never makes an increase before the last dose is tolerated, and without any feverish reaction. The injections are given once every two or three days. The patient is considered as cured when he does not react any more with fever on 0.05 tuberculin. Goetsch had the best results with Koch's old tuberculin. His first principle is never to use it on a feverish patient. The fever must subside before the treatment is commenced. If the fever can no more be subdued, the patient is too far gone for treatment with tuberculin.

(To be continued.)

